# Survival Improvement With Steroid Use for Pulmonary Veno‐Occlusive Disease With the Aid of Pulmonary Vasodilators and Tyrosine‐Kinase Inhibitor, a Retrospective Study

**DOI:** 10.1002/clc.70379

**Published:** 2026-06-22

**Authors:** Mayumi Finger, Kaori Takeuchi, Hanako Kikuchi, Ayumi Goda, Takumi Inami, Yuichi Tamura, Masaharu Kataoka, Toru Satoh

**Affiliations:** ^1^ Department of Cardiovascular Medicine Kyorin University Tokyo Japan; ^2^ Division of Cardiology, Department of Medicine, School of Medicine Keio University Tokyo Japan

**Keywords:** prednisolone, pulmonary hypertension, sorafenib, steroid, veno‐occlusive disease

## Abstract

**Background:**

Steroids are effective in patients with heart failure, connective tissue disease, and interstitial lung disease, which are conditions often associated with Pulmonary veno‐occlusive disease (PVOD). This study assessed the efficacy of steroid treatment combined with pulmonary vasodilators and tyrosine kinase inhibitors in patients with PVOD.

**Hypothesis:**

This retrospective study aims to explore the effect of steroids on patient condition and survival, and the hypothesis that steroids may improve the survival of patients with PVOD was verified.

**Methods:**

The study involved two facilities in Tokyo with the same supervisors. Ten patients were diagnosed with PVOD from April 2006 to May 2018, divided into two categories: the patients not receiving (PNS, *n* = 5) and those receiving steroids (PRS, *n* = 5). The difference in survival between the two groups was evaluated with log‐rank test.

**Results:**

Based on the Kaplan‐Meier curve, median survival length after symptom onset in pulmonary hypertension was 3.3 [1.3–4.3] years (outcome: all died) for PNS (*n* = 5) and 7.1 [5.2–11.0] years (outcome: three were alive, one had lung transplant, and one died) for PRS (*n* = 5). Although steroids did not necessarily improve patients' conditions, PRS had relatively stable conditions for a remarkably extended time compared to PNS.

**Conclusions:**

While the sample set is relatively small and this is a non‐controlled retrospective study, the results indicate that a combination of steroids with pulmonary vasodilators and tyrosine‐kinase inhibitors appears to be a promising treatment, especially for the purpose of extending patient survival until lung transplantation; however, this warrants further large‐scale investigation.

## Graphical abstract

This study aimed to assess the efficacy of steroid treatment with pulmonary vasodilators and tyrosine kinase inhibitors in patients with Pulmonary VenoOcclusive Disease (PVOD). Kaplan‐Meier curve showed that survival of PVOD patients with PSL (PRS) was significantly better with Log rank test (*p* = 0.01) than without PSL (PNS).

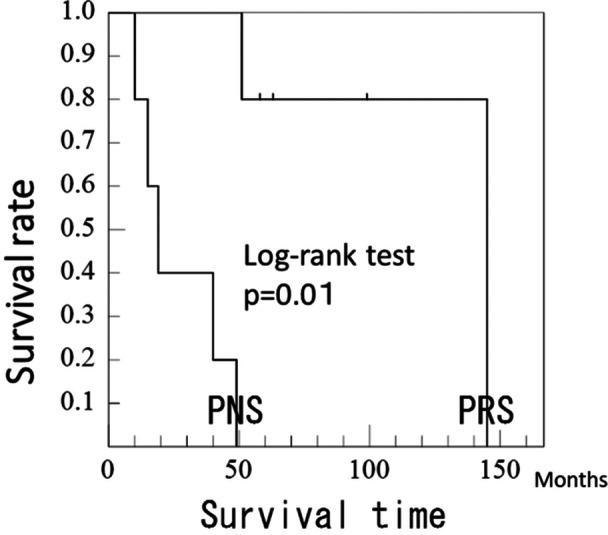

**Abbreviations**
PA: pulmonary artery
PAH: pulmonary artery hypertension
PH: pulmonary hypertension
PNS: patients not receiving steroids
PRS: patients receiving steroids
PSL: prednisolone
PVOD: Pulmonary veno‐occlusive disease
RHC: right heart catheterization

## 1 Introduction

Pulmonary veno‐occlusive disease (PVOD), which is challenging to diagnose, is a rare cause of pulmonary hypertension (PH) that has required numerous reviews of the formal guidelines in the World Symposium on Pulmonary Hypertension [1, 2, 3, 4, 5, 6] different from PAH in small venular or capillary obstruction, severe %DLCO depression, ground‐grass opacity, and interlobular septal thickening due to effective venous decrease or capillary congestion. Although the 2022 PVOD/PCH diagnosis guideline has been published by the Japanese Pulmonary Circulation and Pulmonary Hypertension Society (JPCPHS) [7] no globally accepted guideline for PVOD diagnosis exists. In the Japanese guideline, to definitely diagnose PVOD, in addition to the definite PAH diagnosis, at lease one of following findings of the CT abnormality suggestiong PVOD, severe DLCO decrease, or worseinig of pulmonary edema responding to PA dilators with excluding left heart or pulmonary diseases, are required.

The successful diagnosis of PVOD and distinguishing it from the more common pulmonary artery hypertension (PAH) is critical, as treatment of the latter is largely ineffective on the former. In fact, certain studies believe that PAH treatments have resulted in life‐threatening deterioration [8]. Specifically, administration of vasodilators, including epoprostenol, could cause massive pulmonary edema and thus could be fatal [4, 9]. Nevertheless, diagnosis is just the first step, as there are also no clinically proven treatments specific to PVOD other than lung transplantation. Currently, only individual case studies on PVOD treatment with limited effect have been reported [10, 11]. A case of a 66‐year‐old woman diagnosed as having PVOD who agreed to participate in a study on the use of sorafenib was previously reported by the authors. Although complications such as eruptions occurred during the study, sorafenib was found to improve PVOD from NYHA class IV to class II over 12 months. The study demonstrated that sorafenib may be a potential treatment for PVOD [12]. Case studies from other countries have suggested that diuretics plus bosentan [10] and warfarin [11] may improve the patient's saturation and overall condition for periods of 1–4 years. Anticoagulants are not recommended to be used in PVOD unless their use is beneficial in the specific patients [6]. In 1991, a case study reported on a 28‐year‐old female with PVOD treated with steroids, which resulted in the remission of interstitial pneumonia; however, without PH medication, such treatment may still ultimately lead to fatal disease progression [13]. Similarly, some studies showed a positive effect of steroids in patients with an autoimmune disease associated with PVOD [14, 15, 16]. However, no formal study on the efficacy of steroid treatment for PVOD, specifically when combined with the latest PH medication, has been conducted. Nonetheless, in a report that included eight patients (of which six were believed to have PVOD), treatment was performed at two sites and focused on the use of epoprostenol for 102 to 1063 days (a maximum of 3 years). Results showed improvement, that is, from WHO functional classes III and IV to classes II and III, along with improved oxygen saturation. The treatment was believed to be a bridge until a lung transplant in four of the patients [17]. However, other studies have different conclusions.

This study aimed to assess the efficacy of steroid treatment with pulmonary vasodilators and tyrosine kinase inhibitors in patients with PVOD. The indications for steroid use included the absence of any effective medical therapy, its marginal effect on patients with heart failure (a complication of PVOD) [18], and its effectiveness in the accompanying elements of steroid‐responsive diseases, such as connective tissue disease [19] and interstitial lung disease [13]. Although lung transplantation is the only effective treatment, very few lung transplantations (0.26/one million population) [20] have been conducted in Japan compared to the United States (5.6/one million population) for socio‐ethical reasons [21]. Therefore, some form of medical treatment must be relied on in Japan. Here, the effect of steroid treatment on patients' conditions and ultimately, survival was explored. To confirm the diagnosis of PVOD, the recommended diagnosis categories in the Japanese guideline published by the JPCPHS were used [7], which is similar to the 2022 ESC/ERS Guidelines for the diagnosis and treatment of PH [1].

## 2 Methods

This retrospective cohort study involving two facilities (Kyorin University Hospital and Keio University Hospital) with the same supervisors identified 10 patients (five were treated with steroids and five were not) diagnosed as having PVOD from May 2002 to March 2006 for the non‐steroid group and April 2006 to May 2018 for the steroid group. The standard diagnostic algorithm was followed based on physical examination findings; laboratory data of blood tests; radiographic assessment, such as chest X‐ray and high‐resolution computed tomography scan; pulmonary function test; transthoracic echocardiography and right heart catheterization (RHC), and applied medications. While histological diagnosis has been traditionally required for definitive diagnosis, it is not recommended because of the invasiveness of the procedure [5]. In this study, the recommendations described in the European guidelines [1] and the guidelines for the treatment of PH (JCS2017) were used [22], which are based on various symptoms, clinical data, and some PVOD‐specific radiographic findings, all of which are routinely used for the PVOD diagnosis (Table 1).

**TABLE 1:** Patient diagnosis points.

Patient number	Primary criteria						Secondary criteria			Autopsy
	1. RHC		2a. HRCT		2b. Ruling out	3. Pulmonary congestion due to ERA, NO, PGI_2_ IV				
	mPAP (mmHg)	PAWP (mmHg)	Subpleural thickened septal lines	GGO	ILD and CTD		PaO_2_ (mmHg)	%DLCO	VQ scan^a^	
	25 <	< 15	Yes (Y) or No (N)	Y or N	Y or N	Y or N	< 70	< 55	Y or N	Y or N
PNS1	58	10	Y	Y	Y	N	41	NA	Y	Y
PNS2	52	8	Y	Y	Y	Y	61	34	Y	Y
PNS3	38	5	Y	Y	Y	Y	52	32	Y	Y
PNS4	41	4	Y	N	Y	N	47	16	Y	N
PNS5	80	10	Y	Y	Y	Y	75	61	NA	Y
PRS1	51	8	N	Y	Y	N	66	17	Y	N
PRS2	47	11	N	Y	Y	N	69	24	Y	N^b^
PRS3	43	10	Y	N	Y	N	56	11	Y	N
PRS4	34	8	Y	Y	Y	N	50	30	Y	N
PRS5	27	8	Y	Y	Y	N	60	7	Y	N

*Note:* Following Japanese PVOD diagnosis guideline 2017 (in Japanese). https://minds.jcqhc.or.jp/n/med/4/med0292/G0000960. ① Confirmed diagnosis of PVOD: primary 1 and 2 criteria + pathological diagnosis. ② Diagnosis criteria of PVOD 1: primary 1 and 2 + primary 3 + two or more of secondary criteria. ③ Diagnosis criteria of PVOD 2: primary 1 and 2 + all of secondary criteria. ④ Suspected of having PVOD: primary 1 and 2.

Abbreviations: CTD, connective tissue disease; DLCO, lung carbon monoxide diffusing capacity; ERA, endothelin receptor antagonist; GGO, ground glass opacity; HRCT, high‐resolution computed tomography; ILD, interstitial lung disease; mPAP, mean pulmonary artery pressure; NO, nitrous oxide; PaO_2_, partial pressure of oxygen; PAWP, pulmonary artery wedge pressure; PGI_2_ IV, intravenous prostaglandin I_2_; PNS, patients not receiving steroids; PRS, patients receiving steroids; RHC, right heart catheterization; VQ scan, Ventilation perfusion scan.

^a^ Y: flow defect, N: no significant findings, NA: not available.

^b^ No pathological sample after lung transplantation.

Specifically, each patient's medical history was examined by going through their medical charts and referral letters from other hospitals. Ten patients were divided into two categories. One is the patients not receiving steroids (PNS, *n* = 5). Another is the patients receiving steroids (PRS, *n* = 5). Among the various steroid preparations, all patients were treated with prednisolone (PSL).

During data analysis to assess treatment results, the patients' age was calculated on the day of the occurrence of dyspnea on exertion (PH started day), when PVOD was diagnosed, and on their death (or on May 31, 2018, if still alive). Moreover, data were obtained from their physical examination reports, blood laboratory work, right heart catheterization, pulmonary function test, and pulmonary V/Q scan. For those treated with PSL, the duration of PSL treatment was also evaluated. Survival rates for PNS and PRS groups (May 31, 2018, was the end of the study period for the survivors) were analyzed using the Kaplan‐Meier curve. Background information on individual patients in this study is shown in Table 2. The number and types of administered medications for each patient are listed in Table 2. Sorafenib was administered to two PNS and to three PRS besides the recommended drug combination therapy for PAH [3]. The study protocol of sorafenib administration was approved by the Ethics Committee at Kyorin University Hospital (protocol 09‐06). Written informed consent was obtained before sorafenib and steroid treatment.

**TABLE 2:** Background information and medication.

Patient	Age at diagnosis	Sex	PH diagnosis	Drug				
				ERA	PDE5 I	PGI_2_ IV	Tyrosine KI	PSL dosage^a^
	Years	M or F	Year	Y	Y	Y	Y	Mg
PNS1	73	M	2002	Y	Y	Y		
PNS2	51	M	2004	Y		Y		
PNS3	46	F	2007		Y			
PNS4	52	F	2009	Y	Y	Y	Y	
PNS5	44	F	2010	Y	Y	Y	Y	

PRS1	60	F	2006	Y	Y		Y	30 ￫ 10
PRS2	62	M	2010	Y	Y		Y	30 ￫ 15
PRS3	64	M	2010	Y	Y		Y	30 ￫ 10
PRS4	67	F	2013	Y	Y			30 ￫ 10
PRS5	42	F	2013	Y	Y			30 ￫ 15

Abbreviations: ERA, endothelin receptor antagonist; F, female; M, male; PDE5 I, phosphodiesterase 5 inhibitor; PGI2 DIV, prostaglandin I_2_ intravenous infusion; PGI_2_ IV Y, administered temporally and stopped when pulmonary edema appeared; PNS, patients not receiving steroids; PRS, patients receiving steroids; Tyrosine KI, tyrosine kinase inhibitor; Y, yes.

^a^ Starting dose ￫ maintenance dose.

None of the patients had a connective tissue disease. The patients were tested for various antibodies that indicate a connective tissue disease, and all patients had negative results, including bone morphogenetic protein II. However, one person had Graves' disease (PNS4) since she was young.

### 2.1 Histological and Pathological Diagnosis

Histological diagnosis from biopsy samples was not performed in this study. Postmortem pathological examination confirmed PVOD diagnosis for PNS1, 2, 3, and 5, but not for PNS4. Moreover, pathological examination information was not available for PRS2, who had a double lung transplantation in another country, and for PRS3, who died.

Hemodynamic changes of mPAP and PVR were demonstrated in Figure 3, measured at the onset, after using vasodilators, and after PSL administration only in the PRS group.

### 2.2 Statistical Analysis

Results were reported as median with interquartile range because all data were non‐parametric. Survival of the patients with PVOD in the PNS and PRS groups was evaluated with a Kaplan‐Meier curve; disease onset was determined as the onset of PH symptoms. The difference in survival between the two groups was evaluated with log‐rank test. The duration from PAH to PVOD diagnosis was compared between the groups with the Mann‐Whitney *U* test. The difference in medication use in the two groups was analyzed with a chi‐square test. IBM SPSS Statistics for Macintosh, version 25.0 (IBM Corp., Armonk, NY) was used for all statistical analyses.

## 3 Results

### 3.1 Diagnosis

According to the JPCPHS guideline, PNS1, 2, 3, and 5 had a confirmed diagnosis (primary criteria 1 and 2 with pathological diagnosis), and PNS4 and PRS1, 2, 3, 4, 5 fulfilled the diagnosis criteria of PVOD 2 (primary 1 and 2 + all of secondary criteria) (Table 1).

### 3.2 Background Information

Epoprostenol administration was significantly greater in the PNS group than in the PRS group. Otherwise, background information was not different between the two groups (Table 2).

### 3.3 Prognosis

**FIGURE 1:**
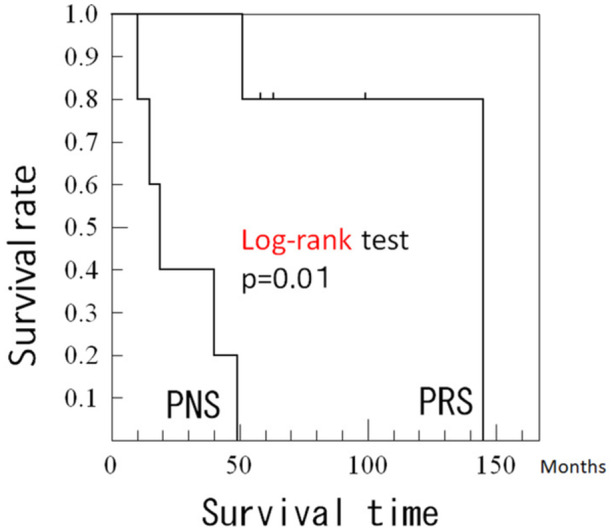
Survival difference of PVOD patients with PSL from without PSL. Kaplan‐Meier curve was depicted to compare the survival of patients using PSL (PRS) with patients not using PSL (PNS). They were significantly different with Log rank test (*p* = 0.01).

**FIGURE 2:**
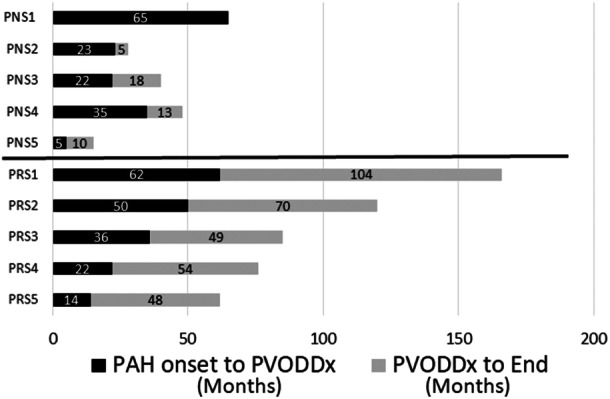
Disease duration of PVOD patients with PSL and without PSL. The duration between the onset of dyspnea due to PAH to PVOD diagnosis and the duration between the diagnosis of PVOD to death (transplantation) or the last followed date were depicted. The patients were aligned in the chronological order. The first patient in the PNS group was difficult to diagnose in the authors' center. The second patient in the PNS was diagnosed at the last stage of the disease. Most of the patients with long duration until diagnosed correctly in PRS group spent the period in the referring hospitals. The duration of the diagnosis of PAH to PVOD were not different between PNS and PRS groups.

The PRS group has a longer survival than the PNS group (Figure 1). Among those treated with PSL (PRS1–5), the median survival length was 7.1 years (interquartile range = 5.2–11.0 years) as of May 2018. Furthermore, PRS2 survived > 5 years and subsequently had a lung transplant, and PRS3, who died, survived > 8 years without a transplant, which is almost 1.5 times the duration of the longest survival in the PNS group; mortality occurred within 3.5 years (median survival length of 3.3 years [interquartile range = 1.3–4.3 years]) in most of the patients in the PNS group. The duration of the symptom (dyspnea) onset in PAH to PVOD diagnosis was not different between the PNS and PRS groups (23 [18, 43] vs. 36 [20, 53] months); however, the duration of PVOD diagnosis to death or the end of study period was significantly shorter in the PNS group (10 [4, 14] months) than in the PRS group (54 [49, 79] months) (Figure 2).

### 3.4 Keyed Clinical Course of the Patients Including NYHA Class on the First Visit and NYHA Class After Treatment

#### 3.4.1 Patients Not Receiving Steroids

PNS1: NYHA3 → 4. The patient was never diagnosed with PVOD before the pathological test.

PNS2: NYHA3 → 4. The patient did not respond to vasodilators and died.

PNS3: NYHA3 → 2. Epoprostenol was started. Hemodynamics improved; however, pulmonary edema aggravated over 4 months, and the patient died.

PNS4: NYHA3 → 2. Despite the improvement with sorafenib treatment, the patient died at 40 months.

PNS5: NYHA3 → 2. At PVOD diagnosis, sorafenib was started, and it kept the patient stable for a few months until she died.

#### 3.4.2 Patients Receiving Steroids

PRS1: NYHA3 → 2. The patient was still alive at the cut‐off date and 10 months from the start of steroid administration.

PRS2: NYHA3 → 2. The patient received a lung transplant abroad 70 months after the start of steroid treatment.

PRS3: NYHA3 → 2. Sorafenib and steroids were added at PVOD diagnosis. Because of difficulty in medication compliance, the patient's health deteriorated and died.

PRS4: NYHA3 → 2. The patient was still alive at the cut‐off date and 54 months after steroid use.

PRS5: NYHA3 → 2. The patient was still alive at the cut‐off date and 47 months after the start of steroid use.

**FIGURE 3:**
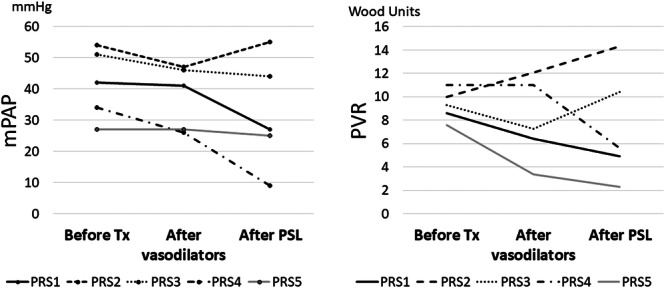
Hemodynamic changes after the treatment including PSL. Three out of five patients in PRS group were decreased in mPAP after PSL administration as same as in PVR.

Details on the PSL dosage are listed in Table 2. PSL was mostly started at 30 mg/day and gradually reduced to 10 or 15 mg while observing clinical changes. Moreover, not much improvement in RHC with PSL use was observed; nevertheless, RHC did not become worse with PSL use (Figure 3).

Sorafenib usage was not different between the two groups.

## 4 Discussion

This retrospective, small‐sized, non‐controlled study showed that steroid therapy, besides pulmonary vasodilators and tyrosine‐kinase inhibitors, could be a promising treatment that extends patient survival until lung transplantation.

Changes in NYHA classes were not so remarkably different between the PSL receiving and not‐receiving groups because both PSL and sorafenib were administered to the both groups and useful in improving their symptoms in NYHA classification, However, their survivals were different manifested in this study.

No previous clinical research reported on the effect of steroids on patients with PVOD [13]. This is the first study to discuss the effect of steroids on patients with PVOD.

PVOD categorization in the PH classification has been changed in every World Symposium on Pulmonary Hypertension [4]. Histological confirmation of PVOD from lung biopsy would be the best definitive diagnosis; however, it is contraindicated in most clinical settings [18]. As biopsy is not recommended, PVOD is difficult to diagnose based on physical and clinical findings or radiographic findings. While a PVOD diagnosis guideline has not been established in the symposium, its categorization has been recommended. In Japan, the JPCPHS created the PVOD/PCH guidelines in 2022 in line with those recommendations [7], which was followed in this study to make the diagnosis as accurate as possible (Table 1). Moreover, although the therapy for PVOD has not been established [4], epoprostenol use has been discussed because of its possible induction of pulmonary edema [4, 9, 23]. Further, the use of imatinib and sorafenib for PVOD has been reported in the aughors' group and in previous studies [12].

The working hypothesis is that for PVOD, which is frequently associated with swelling of thoracic lymph nodes and elevation of an inflammation marker (C‐reactive protein) and is a disease accompanied by blockage of small veins in the lungs that preclude oxygenation, the use of steroids, which typically reduce inflammation and improve blood flow, may have a positive effect on the blockage. For example, PSL, which is an immunosuppressant that is used for autoimmune disorders, may decrease inflammation by reversing increased capillary permeability and suppressing polymorphonuclear leukocyte activity. PSL stabilizes lysosomal membranes and suppresses lymphocytes and antibody production [24]. Thus, some steroids and immunosuppressants may be an effective treatment for PVOD, even in cases without a coexistent inflammatory parenchymal disease and connective tissue/autoimmune disease.

Among the patients who received steroids, PRS4 and PRS5 had a relatively lower mPAP (34 and 27 mmHg, respectively) than the other three patients in spite of similar pulmonary GGOs and decreased DLCO, which could be controlled only by steroids. However, the other three patients with a higher mPAP needed sorafenib in addition to steroids. Thus, steroids could suppress a kind of activity of controlling pulmonary edema in PVOD but could not improve worsened pulmonary artery (PA) hemodynamics, which requires the use of other pulmonary vasodilating medications, including tyrosine‐kinase inhibitors.

Connective tissue and autoimmune diseases are often associated with PVOD [14, 15, 25, 26, 27, 28, 29, 30] and steroids are used to treat interstitial pneumonia complicated with this disease. In the Japanese PVOD guideline, a connective tissue disease is an exclusion criterion for PVOD. None of the patients in this study had a connective tissue disease.

This study has limitations. As the sample size is relatively small and the patients compared were not in a formal controlled clinical trial, It is not stated definitively that steroid is a confirmed treatment that improves patient conditions or results in remission in patients diagnosed as having PVOD. Montani D et al. argued that the annual incidence of PVOD is 0.1–0.2 cases per million in the population [8]. Moreover, as no other studies except a few case papers ever reported the effect of PSL on PVOD, this study including five cases on PSL and five off PSL and comparing the two groups is significant to mention. There seems to be ample evidence that warrants further studies, specifically investigations on the pathological effects of steroid treatment on PVOD. Moreover, based on these findings, steroid treatment could result in an extension of survival that is far beyond any other treatment modality we encountered.

Epoprostenol continuous infusion was instituted in four patients out of five patients in the PNS group but none in the PRS group. This might contribute to the worse prognosis in the former group. Nonetheless, it has been reported that influence of epoprostenol administration to PVOD patients was controversial except in causing absolutely troubled complication of acute pulmonary edema and was beneficial in some patients [31], indicating not the major factor in improvement of the prognosis in this study. The study periods between in the PNS and PRS groups were not overlapped and different, This leads to the argument that the comparison of PNS and PRS groups in prognosis may be impossible. However, no innovative treatment has been developed since the start of this study and background of both groups was not different except epoprostenol and PSL usage, leading to the feasibility of this study.

## 5 Conclusion

This study showed the apparent efficacy of PSL in PVOD treatment; the survival was extended in those who received PSL, which helps while waiting for lung transplantation or new drug discovery. Accordingly, the findings highlight the importance of conducting further studies on steroid treatments combined with pulmonary vasodilators and tyrosine‐kinase inhibitors for PVOD on a larger scale and in a more controlled manner to obtain more statistically valid data.

## Author Contributions

Mayumi Finger was involved in the study design, data collection, data analysis, conceptualization, data curation, investigation, running the software, data validation, visualization, and drafting of the manuscript. Kaori Takeuchi, Hanako Kikuchi, Ayumi Goda, and Yuichi Tamura did data collection, data analysis, conceptualization, data curation, investigation. Takumi Inami and Masaharu Kataoka did the study planning and design, data analysis, conceptualization, data curation, investigation, project administration, data validation, visualization, and drafting of the manuscript. Toru Satoh did the study design, data collection, data analysis, conceptualization, data curation, investigation, project administration, resource allocation, running the software, data validation, visualization, and drafting of the manuscript.

## Funding

The authors have nothing to report.

## Ethics Statement

The authors have nothing to report.

## Conflicts of Interest

The authors declare no conflicts of interest.

## Data Availability

The data that support the findings of this study are available on request from the corresponding author. The data are not publicly available due to privacy or ethical restrictions.
